# Comparison of 1-year outcomes between MAKO versus NAVIO robot-assisted medial UKA: nonrandomized, prospective, comparative study

**DOI:** 10.1186/s43019-020-00030-x

**Published:** 2020-03-12

**Authors:** Chumroonkiet Leelasestaporn, Tomorn Tarnpichprasert, Alisara Arirachakaran, Jatupon Kongtharvonskul

**Affiliations:** 1 Orthopedics Department, Bhumibol Adulyadej Hospital, Bangkok, Thailand; 2 Orthopedics Department, Bumrungrad Hospital, Bangkok, Thailand; 3 Section for Clinical Epidemiology and Biostatistics, Faculty of Medicine, Ramathibodi Hospital, Bangkok, Thailand; 4 Orthopedic Department, Payathai 3 Hospital, Bangkok, Thailand

**Keywords:** MAKO, NAVIO, Robotic surgery, UKA

## Abstract

**Background:**

We have conducted a prospective cohort study with the aim of comparing operative time, intraoperative blood loss, and radiologic and clinical outcomes between imageless (NAVIO) and image-based (MAKO) robot-assisted unicompartmental knee arthroplasty (UKA) for medial compartment osteoarthritis (OA) of the knee.

**Methods:**

A total of 33 patients with medial compartment OA of the knee were prospectively allocated on alternate operative days of their surgery to MAKO (16 patients) or NAVIO (17 patients) robot-assisted UKA. The primary outcome (Knee Society Score [KSS] and Knee Functional Score [KFS]) and the secondary outcomes (intraoperative time of seven steps [registration of hip and ankle, femur and tibia, ligament tension, implant planning, preparation femur, tibia and trial implant], component alignment [coronal and sagittal of femur, tibia implant], blood loss, complications, and revision at 1 year after surgery) were compared between two groups. Statistical significance was set at *P* < 0.05.

**Results:**

Mean KSS measured at baseline and 1 year were, respectively, 70.3 (5.6) and 96.9 (5.7) in the NAVIO group and 72.3 (4.5) and 94.7 (10.01) in the MAKO group. Mean KFS measured at baseline and 1 year were, respectively, 95.5 (7.9) and 99.9 (0.25) in the NAVIO group and 67.3 (7.8) and 99.5 (1.2) in the MAKO group. There were no significant differences for KFS and KSS outcomes (*P* = 0.203 and *P* = 0.457, respectively) between the NAVIO and MAKO groups. Mean operative time and blood loss in the NAVIO versus MAKO robot-assisted UKA groups were 98 min versus 82.5 min and 136.3 ml versus 80 ml, respectively, and these differences were statistically significant. In the MAKO group, the intraoperative time was statistically significantly shorter in registration of hip and ankle center, femur and tibia, femur preparation, and trial implantation compared with the NAVIO group. There were no significant differences of component alignment and radiologic alignment at 1 year between the two groups. No perioperative or delayed complications (infection, periprosthetic fracture, thromboembolism, and compromised wound healing) and revisions were reported in either group.

**Conclusions:**

This study demonstrated that two robotic systems showed no difference in clinical outcomes at 1 year and radiologic alignment of implants, whereas operative time and intraoperative blood loss were found to be less in MAKO robot-assisted UKA.

**Trial registration:**

ClinicalTrials.gov, NCT03954912. Registered on 17 May 2019.

**Level of evidence:**

II

## Introduction

Unicompartmental knee arthroplasty (UKA) offers potential functional advantages over total knee arthroplasty (TKA) [1, 2]. One of the greatest challenges to both uptake of UKA by surgeons and the ultimate success of the surgery has been the technically demanding nature of the surgery. Recent changes in component design, surgical instrumentation, and surgical techniques have led to improved UKA radiographic and clinical outcomes of UKA [3–6]. The changes in surgical instruments that have taken place include systems that allow more accurate flexion–extension gap balancing and more accurate bone preparation. However, despite these improvements in manual instruments, some surgeons have also recently adopted advances in robotic surgery that have led to improved accuracy and alignment of UKA prostheses [6–13]. Currently, there are two semiautonomous systems approved by the U.S. Food and Drug Administration for robot-assisted UKA: an imageless (NAVIO) system [14] and an image-based (MAKO) system [6, 8, 13, 15, 16]. The current semiautonomous systems use different methods to safeguard against inadvertent bone preparation, one by providing haptic constraint beyond which movement of the burr is limited (MAKO: Restoris MCK partial knee implant system; Stryker, Kalamazoo, MI, USA) and the other by modulating the exposure or speed of the handheld robotic burr (NAVIO: Journey UNI unicompartmental knee system; Smith & Nephew, Memphis, TN, USA). These systems also provide real-time quantification of soft-tissue balancing, which may contribute to the reported successful clinical and functional outcomes with semiautonomous systems [17]. The results show that both robot-assisted surgical systems have better intraoperative (surgical time and blood loss) and postoperative (range of motion [ROM], function, complications, and revisions) outcomes and return to activity than conventional UKA [13–16]. However, no randomized controlled trials, systematic reviews, or meta-analyses have compared intraoperative (surgical time, tourniquet time, operative time and blood loss) and postoperative (ROM, function, complications, revisions and return to activity) outcomes of the NAVIO versus MAKO systems in UKA. Therefore, we conducted a prospective cohort study with the aim of comparing operative time, intraoperative blood loss, and radiologic and clinical outcomes between imageless (NAVIO) and image-based (MAKO) robot-assisted UKA for medial compartment osteoarthritis (OA) of the knee. We hypothesized that use of the MAKO system in medial UKA would improve clinical outcomes, including intraoperative (surgical time and blood loss) and postoperative outcomes, compared with the NAVIO system.

## Methods

**Fig. 1:**
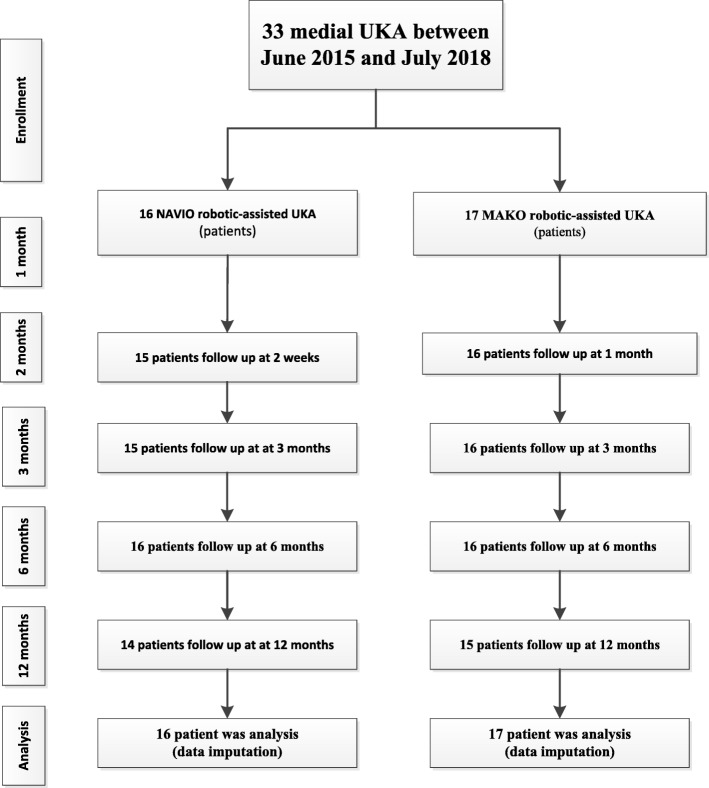
Flowchart of the prospective cohort study on range of motion (ROM), Knee Society Score (KSS), and Knee Functional Score (FS). *UKA* unicompartmental knee arthroplasty

Our study included 33 patients who underwent medial UKA performed by a single senior surgeon (CL) with either the NAVIO or MAKO robot-assisted system between 1 June 2015 and 1 July 2018 at Bhumibol Adulyadej Hospital. This study was approved by the Committee on Human Rights Related to Research Involving Human Subjects at the Bhumibol Adulyadej Hospital under the protocol ID 2/62. All patients had been listed for UKA to treat medial compartment knee OA and were recruited by a research associate (TT). Eligible patients were those deemed suitable for UKA by a senior surgical author (CL), could give informed consent, and were willing to attend the prescribed follow-up. Exclusion criteria included those with ligament insufficiency (anterior cruciate ligament rupture; collateral ligament insufficiency); inflammatory arthritis; a deformity requiring augmentation; neurological movement disorders; pathology of the feet, ankles, hips, or opposite knee causing significant pain or gait alterations; and those who ultimately required TKA. (Valgus greater than 14 degrees and multiple compartment OA were contraindications to UKA.) The 33 patients were allocated for either image-based (MAKO) or imageless (NAVIO) procedures on alternate days of their surgery, with 16 assigned to the NAVIO robot-assisted UKA cohort and 17 to the MAKO robot-assisted UKA cohort (Fig. 1). Four UKA procedures were performed regularly in 1 day. There were no significant demographic differences between the groups (Table 1).

**Table 1:** Characteristics of patients who underwent NAVIO and MAKO robotically assisted unicompartmental knee arthroplasty at baseline, intraoperatively, and 1 year postoperatively

Outcomes	NAVIO robotically assisted UKA (*n* = 16)	MAKO robotically assisted UKA (*n* = 17)	*P* value
Baseline characteristics			
Age, yr, mean (SD)	70.9 (5.9)	71.5 (6.3)	0.573
Sex, *n* (%)			
Male	4 (25.0)	4 (23.5)	0.922
Female	12 (75.0)	13 (76.5)	
Weight, kg, mean (SD)	64.8 (9.5)	62.4 (9.3)	0.479
Height, cm, mean (SD)	157.8 (7.0)	155.1 (4.8)	0.203
BMI, kg/m^2^, mean (SD)	26.0 (3.17)	25.8 (3.3)	0.912
Preoperative ROM in flexion, degrees, mean (SD)	130 (5.5)	129.4 (5.8)	0.768
Preoperative ROM in extension, degrees, median (range)	0 (0–5)	0 (0)	0.07
Preoperative KSS, mean (SD)	70.3 (5.6)	72.3 (4.5)	0.265
Preoperative KFS, mean (SD)	65.5 (7.9)	67.3 (7.8)	0.515
Intraoperative results			
Method of anesthesia			
Epidural anesthesia (%)	14 (87.5)	16 (0.94)	0.509
General anesthesia (%)	2 (12.5)	1 (0.06)	
Tourniquet time, minutes, mean (SD)	113.6 (8.4)	109.4 (18.7)	0.416
All seven surgical step time, min, mean (SD)	98.0 (8.4)	82.5 (11.8)	0.0002
Operative time, min, mean (SD)	134.4 (9.3)	130.4 (20)	0.472
Blood loss, ml, median (range)	136.3 (108–155)	80 (68–132)	0.006
Postoperative results			
Hospital stay, d, mean (SD)	4.8 (1.2)	4.4 (1.1)	0.406
Radiographic postoperative findings			
Acceptable femur varus/valgus angle	16	17	–
Acceptable femur flexion/extension angle	16	17	–
Acceptable tibia varus/valgus angle	16	17	–
Acceptable tibia posteroinferior angle	16	17	–
ROM in flexion at 1 yr, degrees, mean (SD)	130.3 (5.6)	131.2 (6.7)	0.693
ROM in extension at 1 yr, degrees, mean (SD)	0 (0)	0 (0)	–
Knee Functional Score at 1 yr, mean (SD)	99.9 (0.25)	99.5 (1.2)	0.203
Knee Society Score at 1 yr, mean (SD)	96.9 (5.7)	94.7 (10.1)	0.457
Postoperative complications at 1 yr	0	0	–
Revisions at 1 yr	0	0	–

Acceptable femur varus/valgus angle of the femoral component relative to the femur is < 10 degrees varus to < 10 degrees valgus)

Acceptable femur flexion/extension angle of the femoral component relative to the femur is within 15-degree flexion to 0-degree extension

Acceptable tibia varus/valgus angle of the tibial component relative to the tibia is < 5 degrees varus to < 5 degrees valgus

Acceptable tibia posteroinferior tilt of the tibial component relative to the tibia is 7 ± 5 degrees

*Abbreviations: BMI* body mass index, *KFS* Knee Functional Score, *KSS* Knee Society Score, *ROM* range of motion, *UKA* unicompartmental knee arthroplasty

### Surgical technique

**Fig. 2:**
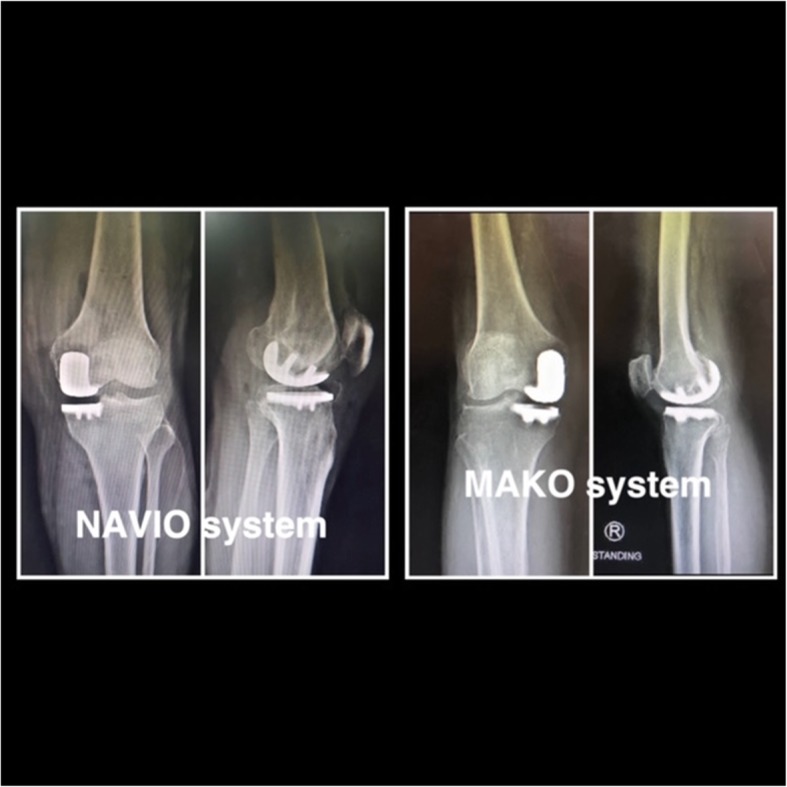
X-rays of postoperative patient (our series) who underwent unicompartmental knee arthroplasty with image-based MAKO system and imageless NAVIO system. This picture show images of two patients who underwent two different procedure

All surgical procedures were performed by the same surgeon (CL) with the patient under either epidural or general anesthesia. A tourniquet was applied and inflated before registration. All patients received 1 g of tranexamic acid at induction. Patients in both treatment groups received a combination of 20 ml of 0.5% bupivacaine, normal saline solution 40 ml, Ketorolac 30 mg (Pfizer, New York, NY, USA), and adrenaline 0.5 ml injected into the joint capsule prior to wound closure. The tourniquet was deflated after wound closure. The implant used was a cemented, fixed-bearing unicompartmental prosthesis with metal-bearing polyethylene (Restoris MCK partial knee implant system; Stryker) in the image-based MAKO robotic system and the Journey UNI Unicompartmental knee system (Smith & Nephew) in the imageless NAVIO system. In the MAKO group, preoperative computed tomography was performed and a 3D computer model of the knee was constructed by a trained technician. A medial parapatellar quadriceps-sparing incision and approach were used, and UKA was performed using the instrumentation in accordance with the operative technique. All three articular compartments and the cruciate ligaments were examined to confirm suitability for UKA. The proper surgical technique of image-based MAKO [15] and imageless NAVIO [18] robot-assisted UKA have been described previously. Figure 2 shows postoperative x-rays of MAKO and NAVIO robot-assisted UKA.

### Postoperative rehabilitation

All patients in both groups followed the same standardized postoperative rehabilitation program, with full weight-bearing and active ROM exercises commenced from the day of surgery. Each physiotherapy session lasted 25 min in total, and all rehabilitation was performed by the same multidisciplinary team in both treatment groups. Patients were discharged after adequate pain control, knee flexion to a minimum of 90 degrees, independent mobilization with the use of crutches, and independent ascent and descent of stairs. All study patients were discharged to home. No patients were discharged to a rehabilitation center or other skilled nursing facility.

### Clinical assessment

Preoperative data regarding age, sex, body mass index, and ROM were recorded. Patients were followed up postoperatively at 12 months. Full hospital and clinic medical record review of demographic, preoperative, intraoperative (mean all 7 surgical step time and tourniquet time), intraoperative blood loss (visible blood loss were observed before tourniquet release), and postoperative knee score measurements (Knee Functional Score [KFS] and Knee Society Score [KSS]) was performed. The primary outcomes of interest were KSS and KFS. Mean intraoperative time was divided into seven steps (registration of hip and ankle, femur and tibia, ligament tension, implant planning, preparation femur, tibia, and trial implant). The KSS questionnaire includes seven items and has a maximum score of 100 (the higher the score, the better the function), and the KFS questionnaire includes four subgroups and has a maximum score of 100 (the higher the score, the better the function). These were used by a well-trained research assistant (orthopedic resident and staff) at baseline and months 1, 2, 3, 6, and 12 after surgery. Any postoperative complications were recorded, such as deep vein thrombosis, infection, loosening of implants, fractures, lateral compartment arthritis, and dislocations of the polyethylene component.

### Radiological assessment

Radiographic analysis of preoperative and postoperative images evaluating sagittal and coronal alignment and component positioning was performed by two fellowship-trained orthopedic surgeons with significant experience in UKA surgery. Postoperative femoral and tibial components were measured using radiographs 1 week and 12 months postoperatively, according to a previous report [19, 20]. Inter- and intraobserver reliabilities were assessed using the kappa statistic.

### Statistical analysis

The sample size was calculated to detect a mean difference in KSS (0–100) between MAKO and NAVIO robot-assisted UKA. From the pilot study, the mean and standard deviation (SD) of KSSs in UKA with MAKO robot-assisted and NAVIO robot-assisted UKA were 91.9 (5.0) and 89.6 (4.8), respectively. Type I error, power of test, and ratio of the treatment groups were set at 0.05, 0.80, and 1:1, respectively. The estimated sample size was 12 for each group in order to detect the minimal clinically significant mean difference of KSS of 6.1 units [21]. Loss to follow-up was estimated to be 20%, which yields a required sample size of 15 patients per group.

Data were described using frequencies for categorical data and mean (SD) or median (range) as appropriate for continuous data. The baseline characteristics of the patients and cointerventions were compared between the two intervention groups using the chi-square test (or exact test when appropriate) and *t* test for categorical data and continuous data, respectively. Continuous outcomes were the intraoperative time of seven steps, with KSS and KFS compared between intervention groups using a two-sample *t* test. Secondary analyses were done using a mixed linear regression analysis with a hierarchical approach. A *P* value < 0.05 was considered statistically significant. All analyses were performed using STATA version 15.0 software (StataCorp, College Station, TX, USA) [22].

## Results

### Patient characteristics

Clinical follow-up of 12 months was available in 33 knees (33 patients), comprising 16 NAVIO and 17 MAKO robot-assisted UKA procedures (Fig. 1). There were no significant differences of preoperative demographics, clinical scores, and method of anesthesia (Table 1). All baseline characteristics were also comparable between treatment groups. Patient compliance with the allocated treatment and follow-up was 100% in both groups. Radiographic findings revealed no differences between the two groups (Tables 1 and 2).

**Table 2:** Mean differences and 95% confidence intervals of intraoperative time compared between NAVIO and MAKO robotically assisted unicompartmental knee arthroplasty

Surgical steps	NAVIO robotically assisted UKA (*n* = 16)	MAKO robotically assisted UKA (*n* = 17)	Mean difference and 95% confidence interval	*P* value
Hip and ankle center registration	5.6 (3.9, 7.2)	2 (0.6, 3.4)	3.6 (1.5, 5.7)	0.001
Registration of femur and tibia	8.4 (6.8, 10.0)	5.4 (4.0, 6.8)	3.0 (0.9, 5.1)	0.005
Registration of ligament tension	1.9 (0.2, 3.5)	2.8 (1.4, 4.2)	− 0.9 (− 3.1, 1.2)	0.389
Implant planning	6.1 (4.5, 7.7)	5.2 (3.8, 6.5)	0.9 (− 1.2, 3.0)	0.398
Femoral preparation	11.5 (9.9, 13.1)	7.6 (6.2, 9.0)	3.9 (1.8, 6.0)	< 0.001
Tibial preparation	8.2 (6.6, 9.8)	8.0 (6.6, 9.4)	0.2 (−1.9, 2.3)	0.843
Trial implantation	8.9 (7.2, 10.5)	5.4 (4.0, 6.8)	3.4 (1.3, 5.6)	0.001
Total operative time, min, mean (SD)	98.0 (8.4)	82.5 (11.8)	15.4 (8.1, 22.8)	0.0002

*UKA* unicompartmental knee arthroplasty

#### Seven steps of intraoperative time

Seven steps of intraoperative time were plotted by treatment and time, which indicated that the MAKO group had faster time in all seven steps than the NAVIO group (Table 3). The mixed-effect regression model indicated that the MAKO group scored 14.55 (95% confidence interval, 11.33, 17.77), significantly higher than the NAVIO group (Table 3).

**Table 3:** Mean Knee Society and Knee Functional scores compared between NAVIO and MAKO robotically assisted unicompartmental knee arthroplasty at follow-up time points 1, 2, 3, 6, and 12 months

Follow-up time	NAVIO robotically assisted UKA (*n* = 16)	MAKO robotically assisted UKA (*n* = 17)	Mean difference and 95% confidence interval	*P* value
Knee Society Score				
1 month	91.9	89.6	2.2 (− 0.3, 4.7)	0.081
2 months	95.9	94.6	1.3 (− 1.2, 3.8)	0.313
3 months	97.3	96.4	0.8 (− 1.7, 3.3)	0.511
6 months	98.6	98.9	− 0.32 (− 2.8, 2.2)	0.802
12 months	99.9	99.5	0.41 (− 2.1, 2.9)	0.749
Knee Functional Score				
1 month	80.9	89.8	− 8.8 (− 13.6, − 4.0)	< 0.001
2 months	86.3	92.6	− 6.4 (− 11.2, − 1.6)	0.009
3 months	91.3	93.8	− 2.6 (− 7.4, 2.2)	0.294
6 months	94.4	94.4	− 0.04 (− 4.8, 4.8)	0.988
12 months	96.9	94.7	2.2 (− 2.6, 7.0)	0.376

*UKA* unicompartmental knee arthroplasty

#### Mean Knee Society Score

Mean KSS was plotted by treatment and time, which indicated increasing KSS after surgery in both groups. Applying the mixed-effect regression model indicated no significant difference between the two groups at each time point (Table 4).

**Table 4:** Postoperative mechanical axis and mean value of each angle (coronal and sagittal of femur, tibia implant) in the two study groups

Angle	NAVIO robotically assisted UKA (*n* = 16)	MAKO robotically assisted UKA (*n* = 17)	*P* value
Mechanical axis			
Preoperative	165.7 (7.5)	166.7 (5.3)	0.659
Postoperative	180.1 (2.2)	179.7 (1.5)	0.544
Femoral			
Coronal	90.1 (0.9)	90.2 (0.7)	0.723
Sagittal	90.2 (1)	89.7 (0.4)	0.066
Tibial			
Coronal	90 (0.5)	89.7 (1)	0.289
Sagittal	− 5.8 (1)	− 6.1 (1)	0.396

< 180 of HKA (mechanical axis) and < 90 of femoral, tibial coronal means varus

< 90 of femoral sagittal means flexion

Negative value of tibial sagittal means postslope

*UKA* unicompartmental knee arthroplasty

#### Mean Knee Functional Score

Mean KFS was plotted by treatment and time, which indicated increasing KFS after surgery in both groups. Applying the mixed-effect regression model indicated significantly lower KFSs of 8.8 and 6.4 between the two groups at 1- and 2-month follow-up (Table 4). No perioperative or delayed complications (infection, periprosthetic fracture, thromboembolism, and compromised wound healing) and revisions were reported in either group.

## Discussion

In this prospective cohort study, we compared the radiologic and clinical outcomes between imageless (NAVIO) and image-based (MAKO) robot-assisted UKA at 1 year. Our hypothesis was that use of the MAKO system in medial UKA could improve clinical outcomes, which include intraoperative (surgical time and blood loss) and postoperative outcomes, compared with use of the NAVIO surgical system, and this study showed no significant differences of KFS and KSS at 1 year. However, intraoperative time of seven steps (registration of hip and ankle, femur and tibia, ligament tension, implant planning, preparation femur, tibia, and trial implant) showed that the MAKO system required statistically significantly less time (about 16 min) than the NAVIO system. In addition, intraoperative blood loss with the MAKO system was statistically significantly less by about 56 ml than with the NAVIO system. No perioperative or delayed complications (infection, periprosthetic fracture, thromboembolism, and compromised wound healing) and revisions were reported in either group.

Our results show that the image-based system (MAKO) resulted in a significant decrease in operative time (registration to trial implant) and blood loss and an increase in knee function compared with the imageless system (NAVIO) in robot-assisted UKA. The reasons for this are undetermined in this study, but a possible explanation would be as follows. First, the MAKO robotic system uses an image-based procedure that is much faster than the NAVIO system, which may lead to less blood loss. Second, the MAKO system allows surgery to be tailored to the patient’s anatomy, with more accurate reconstruction of the joint surfaces and the potential for more natural knee kinematics. Third, the use of a MAKO robotic arm-mounted irrigated burr rather than a traditional high-speed saw blade may prevent excessive heat-associated bone necrosis and might facilitate more minimal bone resection, both of which may lead to less postoperative pain and blood loss and better function [16]. However, the differences of KFS at 1–2 months postoperatively were not clinically important, because clinical outcomes were similar with progression of time. As most patients still have pain, swelling, limited ROM, or related symptoms at 1–2 months after surgery, outcomes at this time may not reflect the final function of the patients.

Although MAKO and NAVIO robot-assisted UKA is not a new technique in arthroplasty and there have been many studies of MAKO or NAVIO robot-assisted UKA, there have been no studies comparing MAKO versus NAVIO robot-assisted UKA. To the best of our knowledge, this study is the first study that assessed any steps in operative time, KSS, KFS, and complications of UKA with MAKO compared with NAVIO robot-assisted UKA. The follow-up was reasonably high at a rate of 100% in both groups. We applied a subgroup analysis by adjusting unequal baseline characteristics of patients in both groups, thus minimizing bias.

### Limitations

Our study has some limitations. First, the subjects in our study were not randomized into the two groups, because the baseline characteristics (severity of arthritis, preoperative KSS and KFS) would have been evaluated during surgery, and then the patients were included in either one group or another. However, we allocated the patients according to the date of surgery and blinded the evaluation of the intraoperative time, KSS, KFS, and complications of the patients in both groups. Second, this is a small cohort with 16 patients in the NAVIO group and 17 patients in the MAKO group. Only 33 were eligible for statistical review. The sample size calculation was computed to assess primary outcomes between groups but may not be generalized to assess secondary outcomes; therefore, statistical insignificance might be due to the risk of type II error. Third, this study was done with a short follow-up period (short-term [1-year] postoperative outcomes of UKA); thus, long-term effects of both techniques for UKA are still in question. Last, we considered only the KSS, KFS, and complication outcomes. Further study should assess outcomes such as costs of the operation and postoperative satisfaction and quality of life to compare MAKO and NAVIO robot-assisted UKA.

## Conclusion

This study demonstrated that two robotic systems showed no differences in clinical outcomes at 1 year and radiologic alignment of implants, whereas operative time and intraoperative blood loss were found to be less in MAKO robot-assisted UKA.

## Data Availability

Not applicable.
